# Supplementary material for: Validation of a Questionnaire Developed to Evaluate a Pediatric eHealth Website for Parents
Source: Int J Environ Res Public Health. 2020 Apr 13;17(8):2671. doi: 10.3390/ijerph17082671 (PMC7215976; doi:10.3390/ijerph17082671)
Supplement: Supplementary file 1 [file ijerph-17-02671-s001.pdf]

Appendix. Supplementary Files A1-A12.

All appendix sections must be cited in the main text. In the appendixes, Figures, Tables, etc. should be labeled starting with 'A', e.g., Figure A1, Figure A2, etc.

[**Supplementary file A1].**

Checklist for Reporting Results of Internet E-Surveys (CHERRIES).

**Checklist for Reporting Results of Internet E-Surveys (CHERRIES).**	
**Design.**	
**Describe survey design.**	The target population of this questionnaire were the users of **notodoespediatria.com**. Our sample was a convenience sample.
**Institutional Review Board approval and informed consent process.**	
IRB approval.	This study used secondary data sources and did not collect personal information. IRB approval was not necessary.
Informed consent.	There was an informed consent at the beginning of the questionnaire. Participants were informed of the purpose of the study that no personal information was going to be collected. They were also informed of the length of the questionnaire.
Data protection.	The questionnaire did not collect personal information, so additional mechanisms to protect unauthorised access to those provided by the platform (Google Forms) were not needed.
**Development and pre-testing.**	
Development and testing.	The questionnaire was developed by the five authors of the research, following the CHERRIES checklist and ensuring usability and technical functionality. For external validation of contents, the electronic questionnaire was reviewed by seven external expert reviewers. For internal validation, statistical analysis were performed.
**Recruitment process and description of the sample having access to the questionnaire.**	
Open survey versus closed survey.	The questionnaire was an open survey.
Contact mode.	The initial contact with the potential participants was made on the Internet, mainly in notodoespediatria.com.
Advertising the survey.	Advertising the survey. The survey was not announced or advertised in other webs, but users of notodoespediatria.com were invited to participate trough invitations posted via social media (Facebook, Twitter).
**Survey administration.**	
Web/E-mail.	The e-survey was posted on the Web site that was going to be evaluated by the users (**notodoespediatria.com**).

Context.	The survey was developed in Google Forms platform and posted in **notodoespediatria.com**, a web with paediatric resources for parents with 338 posts about symptoms, illnesses and well-child care advices, written by a paediatrician with evidence based information.
Mandatory/voluntary.	Completing and submitting the survey was voluntary.
Incentives.	No incentives were offered.
Timeframe.	The survey was open for 8 weeks.
Randomization of items or questionnaires.	No randomization of items was performed.
Adaptive questioning.	No adaptive questioning was needed.
Number of Items.	The questionnaire included 67 items: 26 demographical items and 41 qualitative items.
Number of screens (pages).	The questionnaire had 10 pages, including initial informed consent.
Completeness check.	All items of the questionnaire were stated as required. Therefore, to submit a questionnaire all items should be answered. By consensus, the authors concluded that only twenty one items of the questionnaire should include a «not applicable» or «rather not say» option.
Review step.	Respondents were able to review and change their answers through a Back button.
**Response rates.**	
Unique site visitors.	Notodoespediatria.com had 98,577 unique visitors during the eight weeks the survey was open.
View rate (Ratio unique site visitors/unique survey visitors).	Google Forms did not provide the number of unique visitors of the first page of the survey.
Participation rate (Ratio unique survey page visitors/agreed to participate).	Google Forms did not provide this data.
Completion rate (Ratio agreed to participate/finished survey).	Of the 98,577 unique visitors of **notodoespediatria.com** during the eight weeks the survey was open, 516 questionnaires were submitted. Therefore, an estimation of the completion rate is 0.52% of the unique visitors of the web.
**Preventing multiple entries from the same individual.**	
Cookies used.	No cookies were used to assign a unique user identifier to each client computer. As personal information was not collected, we could not prevent duplicates entries.
IP check.	The IP address of the client computer was not collected nor used to identify potential duplicate entries from the same user.
Log file analysis.	Other techniques to analyse the log file for identification of multiple entries were not used.

Registration.	No registration was needed.
**Analysis.**	
Handling of incomplete questionnaires.	Only completed questionnaires could be submitted.
Questionnaires submitted with an atypical timestamp.	Google Forms only provided a timestamp.
Statistical correction.	No statistical correction was needed.

[Supplementary file A2].

English translation of the original Spanish questionnaire.

(Please consider that translation of the items into English does not replace a trans-cultural adaptation of the instrument).

Observational items (26 items).

Demographical data (6 items).

1. What is your gender?
—Man.
—Woman.
2. What is your age? (In years)
—Write a number.
3. What is your educative level?
—Primary education.
—Secondary education.
—University education.
—Post-graduate studies.
—Other.
4. What is your total household income?
—Less than 10.000€ per year.
—11.000€ - 25.000€ per year.
—26.000€ - 50.000€ per year.
—51.000€ - 75.000€ per year.
—More than 75.000€ per year.
5. Where do you live?
—Spain.
—Other European country.
—North America.
—Central/ South America.
—Africa.
—Asia.
—Oceania.
6. Which geographic area do you live?
—Urban.
—Rural.

About your children (5 items).

7. How many children do you have?
—0.
—1.
—2.
—3 or more.

8. How old is your youngest child?
[Age in years].
9. Has any of your children a chronic disease?
—Yes.
—He is being studied.
—No.
10. Has any of your children a severe disease?
—Yes.
—He is being studied.
—No.
11. How often do you go to your primary care center to see a paediatrician, with your youngest child?
—0 - 1 visits per year.
—2 - 3 visits per year.
—4 - 7 visits per year.
—7 - 10 visits per year.
—More than 10 visits per year.

Use of the Internet (10 items).

12. Which device do you use to access the Internet?
—Computer.
—Smartphone.
—Tablet.
—Any of them.
13. Can you access the Internet from your phone?
—Yes.
—No.
—I don't know.
14. How often do you use the Internet?
—Several times each day.
—Once a day.
—Each two or three days.
—One or two times per week.
—Once time a month, or less.
15. Dou you use the Internet for health searches?
—Yes.
—No.
16. When you use the Internet for health searches for you, the results have helped you to decide if to go, or not, to see a doctor?
—Yes.
—No.
—I don't know.
17. When you use the Internet for health searches for your children, the results have helped you to decide if to go, or not, to see a paediatrician?
—Yes.
—No.
—I don't know.
18. How do you search for health information, in the Internet?
—Using general searching engines, like Google.
—I go straightly to the webs that I want to consult.
—I use social media, forums or others.
19. How much do you trust in the Internet for health consultations about you?
—Much.

—Quite.
—A bit.
—Not too much.
—Nothing.
20. How much do you trust in the Internet for health consultations about your children?
—Much.
—Quite.
—A bit.
—Not too much.
—Nothing.
21. Do you feel capable of finding paediatric health information, in the Internet?
—Yes, very much.
—Yes, quiet capable.
—A bit capable.
—Not too much.
—Nothing at all.

Use of [Web name blinded for review] (5 items).

22. How did you find the web notodoespediatria?
—Using general searching engines (Google, Yahoo or other).
—Using social media (Facebook, Twitter, other).
—A friend recommended it using Whatsapp, email, SMS or other.
—A friend recommended it verbally.
—A doctor recommended it.
—Two or more of these options.
—None of these options.
23. Which devide do you usually use to access to **[Web name blinded for review]**?
—Computer.
—Smartphone.
—Tablet.
—Any of them.
24. How long did you know **[Web name blinded for review]**?
—Less than a month.
—For 1 - 12 months.
—More than 12 months.
25. How many times have you consulted **[Web name blinded for review]**?
—1 - 5 times.
—5 - 10 times.
—10 - 20 times.
—More than 20 times.
26. The first time you consulted **[Web name blinded for review]**, what where you looking for?
—Information, before consulting a paediatrician.
—Information, about a symptom, before going to an emergencies center.
—To understand something I did not understand after visiting a paediatrician.
—To extend the information that I had received with a paediatrician.
—Information about well-child.
—Other.

Qualitative items (41 items).

Evaluation of technical aspects (5 items).

27. Do you think the visual aspect of **[Web name blinded for review]** is pleasant?
—Very pleasant.
—Quite pleasant.

—A bit pleasant.
—Not too much pleasant.
—Not pleasant at all.

28. If you have used smartphone or tablet, do you think **[Web name blinded for review]** has adapted well to those devices?
—Very well.
—Quite well.
—Not very well.
—Very bad.
—I don't know.

29. Did you find **[Web name blinded for review]** easy to use?
—Very easy.
—Quite easy.
—A bit easy.
—Not too easy.
—Not easy at all.

30. Do you think that the information provided in **[Web name blinded for review]** is easy to understand?
—Very easy.
—Quite easy.
—A bit easy.
—Not too easy.
—Not easy at all.

31. Have you experienced technical problems when using **[Web name blinded for review]**?
—Yes.
—No.

[Web name blinded for review] as a complement of medical consultations (7 items).

32. When using **[Web name blinded for review]**, did you find that the information provided was useful?
—Always.
—Frequently.
—Sometimes.
—Few times.
—Never.

33. The information of **[Web name blinded for review]** has helped you to get more information, before going to see a paediatrician?
—Yes.
—No.
—I don't know.

34. The information of **[Web name blinded for review]** has helped you to understand the information given by a paediatrician?
—Yes.
—No.
—I don't know.

35. Do you feel more confident, consulting the information provided in **[Web name blinded for review]**, as a complement of the information provided by your paediatrician?
—Yes.
—No.
—I don't know.

36. As a consequence of visiting **[Web name blinded for review]**, have you ever avoided a visit to your paediatrician?
—Yes, in two or more occasions.

—Yes, at least in one occasion.
—No, never.
—I don’t know.

37. As a consequence of visiting **[Web name blinded for review]**, have you ever avoided a visit to emergency?
—Yes, in two or more occasions.
—Yes, at least in one occasion.
—No, never.
—I don’t know.

38. Do you think that similar webs, in the long term, can substitute physical consultations?
—Very probable.
—Quiet probable.
—It is possible.
—Not very probable.
—Sure not.

Utility of the web (10 items).

39. Have you ever have a decision, about your children’s health, based on the information provided by **[Web name blinded for review]**?
—Yes.
—No.
—I don’t know.

40. Have you ever followed any of **[Web name blinded for review]** advices?
—Yes.
—No.
—I don’t know.

41. As a consequence of visiting **[Web name blinded for review]**, have you ever decided to go to see a paediatrician?
—Yes.
—No.
—I don’t know.

42. As a consequence of visiting **[Web name blinded for review]**, have you ever decided not to go to see a paediatrician?
—Yes.
—No.
—I don’t know.

43. After visiting **[Web name blinded for review]**, would you have preferred a physical medical consultation?
—Yes.
—No.
—I don’t know.

44. Was **[Web name blinded for review]** useful?
—Very useful.
—Quite useful.
—A bit useful.
—Not very useful.
—Not useful at all.

45. Do you think that **[Web name blinded for review]** inspires trust?
—Very much.
—Quite.
—A bit.
—Not very much.
—Not at all.

46. Do you think that **[Web name blinded for review]** is more trustworthy than other health webs you could have visited?

—Yes.

—No.

—I don't know.

47. Do you think that webs like **[Web name blinded for review]** contribute to improve children's health?

—Yes.

—No.

—I don't know.

48. Do you feel more confident using webs like **[Web name blinded for review]**?

—Yes.

—No.

—I don't know.

Well-child section (6 items).

49. Have you used the well-child section?

—Yes.

—No.

—I don't know.

50. Has the well-child section answered your questions?

—Yes.

—No.

—I don't know.

51. As a consequence of using the well-child section, have you ever avoided a visit to your paediatrician?

—Yes.

—No.

—I don't know.

52. As a consequence of using the well-child section, have you ever avoided a visit to emergency?

—Yes.

—No.

—I don't know.

53. Do you think that everyday handling with your children has improved thanks to the advices of the well-child section?

—Yes.

—No.

—I don't know.

54. Do you feel more confident when handling with your children thanks to the advices of the well-child section?

—Yes.

—No.

—I don't know.

Global evaluation of the web (13 items).

55. Do you think the visual aspect of **[Web name blinded for review]** is pleasant? (1= I completely disagree; 5 = I absolutely agree).

[1-5 points scale].

56. Do you think **[Web name blinded for review]** is easy to use in a computer? (1= I completely disagree; 5 = I absolutely agree).

[1-5 points scale].

57. Do you think **[Web name blinded for review]** is easy to use in a smartphone or a tablet? (1= I completely disagree; 5 = I absolutely agree).

[1-5 points scale].

58. Do you think **[Web name blinded for review]** is written in an easy to understand language? (1= I completely disagree; 5 = I absolutely agree).

[1-5 points scale].

59. Do you think **[Web name blinded for review]** has a wide variety of topics? (1= I completely disagree; 5 = I absolutely agree).

[1-5 points scale].

60. Do you think that searching for information in **[Web name blinded for review]** is easy? (1= I completely disagree; 5 = I absolutely agree).

[1-5 points scale].

61. Do you think that **[Web name blinded for review]** inspires trust? (1= I completely disagree; 5 = I absolutely agree).

[1-5 points scale].

62. How do you rate, from 1 to 5 points, that **[Web name blinded for review]** is an open access web? (1 = very bad; 5 = very good).

[1-5 points scale].

63. How do you rate, from 1 to 5 points, that **[Web name blinded for review]** is a free access web? (1 = very bad; 5 = very good).

[1-5 points scale].

64. Would you recommend **[Web name blinded for review]** to other parents? (1= absolutely; 5 = absolutely not).

[1-5 points scale].

65. Have you recommended **[Web name blinded for review]** to other parents?

—Yes.

—No.

—I don't know.

66. Do you think that similar webs for other medical specialities would be useful?

—Yes.

—No.

—I don't know.

67. What is your final global evaluation of **[Web name blinded for review]**? (1 = very bad; 10 = very good).

[1-10 points scale].

[Supplementary file A3].

Demographic Characteristics of Survey Respondents.

**Demographic Characteristics of Survey Respondents.**		
**Respondent characteristics**		
**Age of respondent.**		**S.D.**
Mean age.	38.81	6.06
Oldest	58	
Youngest	19	
**Age of respondent's youngest child.**		**S.D.**
Mean.	4.62	4.00
Oldest.	19	
Youngest.	0	

[Supplementary file A4].

Factorial analysis. Descriptive statistical data.

**Factorial analysis. Descriptive statistical data.**		
**N = 516**	**Mean.**	**S.D.**
Item 1.	4.22	.694
Item 2.	4.28	.742
Item 3.	4.28	.643
Item 4.	2.03	.100
Item 5.	1.80	.979
Item 6.	1.64	.855
Item 7.	2.91	.313
Item 8.	2.81	.504
Item 9.	2.39	.891
Item 10.	2.54	.735
Item 11.	2.36	.748
Item 12.	2.45	.718
Item 13.	4.62	.591
Item 14.	4.79	.488

[Supplementary file A5].

Items in Spanish original form and English translation.

Items in Spanish original form and English translation.		
**Item.**	**Spanish original item.**	**English translation.**
Item 1.	¿Le ha resultado agradable el aspecto visual de la web?	Do you think the visual aspect of notodoespediatria is pleasant?
Item 2.	De haber usado smartphone o tablet, ¿cree que «No todo es pediatría» se ha adaptado bien a estos dispositivos?	If you have used smartphone or tablet, do you think notodoespediatria has adapted well to those devices?
Item 3.	¿Le ha resultado fácil navegar por «No todo es pediatría»?	Did you find notodoespediatria easy to use?
Item 4.	¿El uso de «**[Web name blinded for review]**» le ha evitado alguna visita a su pediatra?	As a consequence of visiting **[Web name blinded for review]**, have you ever avoided a visit to your paediatrician?
Item 5.	¿El uso de «**[Web name blinded for review]**» le ha evitado alguna visita a Urgencias?	As a consequence of visiting **[Web name blinded for review]**, have you ever avoided a visit to emergency?
Item 6.	¿Alguna vez ha decidido no acudir al médico o pediatra como consecuencia de lo que ha leído en «**[Web name blinded for review]**»?	As a consequence of visiting **[Web name blinded for review]**, have you ever decided not to go to see a paediatrician?

Item 7.	¿« [**Web name blinded for review**]» le inspira confianza?	Do you think that [**Web name blinded for review**]inspires trust?
Item 8.	¿Se siente usted más seguro al disponer de webs como «[**Web name blinded for review**]»?	Do you feel more confident using webs like [**Web name blinded for review**]?
Item 9.	¿Ha utilizado las pestañas de consejos para el niño sano?	Have you used the well-child section?
Item 10.	¿Le han resuelto dudas los consejos para niño sano?	Has the well-child section answered your questions?
Item 11.	¿Cree que el manejo diario de sus hijos ha mejorado gracias a los consejos de niño sano?	Do you think that everyday handling with your children has improved thanks to the advices of the well-child section?
Item 12.	¿Se ha sentido mas seguro en el manejo de sus hijos gracias a los consejos de niño sano?	Do you feel more confident when handling with your children thanks to the advices of the well-child section?
Item 13.	¿Cómo valora, de 1 a 5 puntos, que [**Web name blinded for review**]sea de acceso abierto?	How do you rate, from 1 to 5 points, that [**Web name blinded for review**]is an open access web?
Item 14.	¿Cómo valora, de 1 a 5 puntos, que [**Web name blinded for review**]sea gratuita?	How do you rate, from 1 to 5 points, that [**Web name blinded for review**]is a free access web?

[Supplementary file A6].

Total explained variance.

Total explained variance.			
**Component.**	**Total.**	**% of variance.**	**Accumulated %.**
1	4.342	31.012	31.012
2	2.053	14.663	45.674
3	1.942	13.873	59.547
4	1.085	7.749	67.297
5	1.034	7.382	74.679
6	.586	4.188	
7	.570	4.073	
8	.528	3.774	
9	.426	3.046	
10	.403	2.876	
11	.342	2.441	
12	.286	2.040	
13	.220	1.569	
14	.184	1.313	

Extraction method: principal factor analysis.

[Supplementary file A7].

Commonalities.

**Commonalities.**	**Initial.**	**Extraction.**
Item 1.	1.000	.659
Item 2.	1.000	.630
Item 3.	1.000	.713
Item 4.	1.000	.805
Item 5.	1.000	.733
Item 6.	1.000	.660
Item 7.	1.000	.774
Item 8.	1.000	.751
Item 9.	1.000	.805
Item 10.	1.000	.796
Item 11.	1.000	.772
Item 12.	1.000	.725
Item 13.	1.000	.791
Item 14.	1.000	.840

Extraction method: principal factor analysis.

[Supplementary file A8].

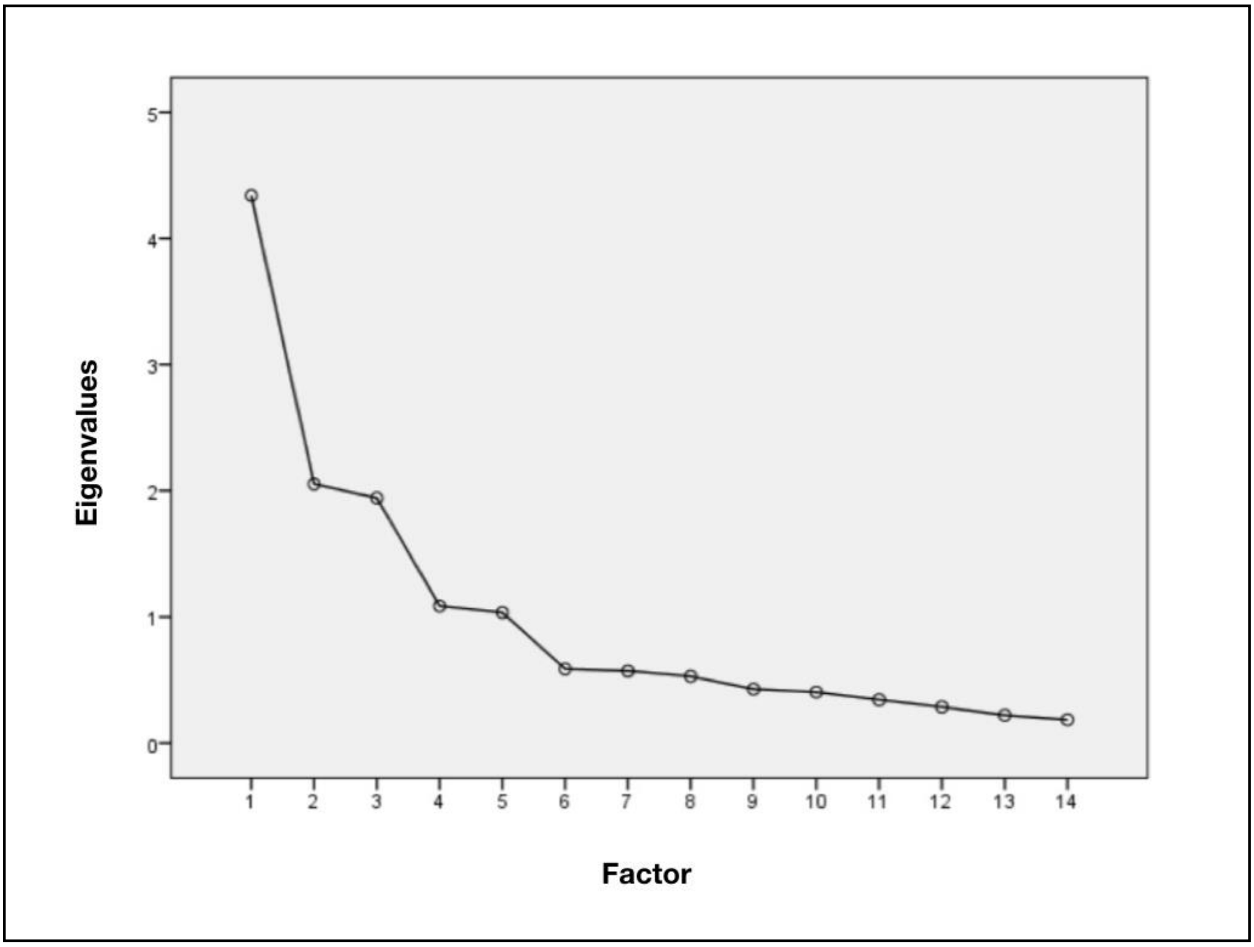

Parallel analysis.
Extraction method: principal factor analysis.

[Supplementary file A9].

Confirmatory factor analysis. Maximum likelihood estimation method.

**Confirmatory factor analysis. Maximum likelihood estimation method.**	
**Notes for model. Computation of degrees of freedom (Default model).**	
Number of distinct sample moments.	105
Number of distinct parameters to be estimated.	38
Degrees of freedom (105 - 38).	67
**Result (Default model). Minimum was achieved.**	
Chi-square.	290.438
Degrees of freedom.	67
Probability level.	0.000
Estimates (Group number 1 - Default model).<br>Scalar Estimates (Group number 1 - Default model).<br>Maximum Likelihood Estimates.	

[Supplementary file A10].

Confirmatory factor analysis. Regression Weights, standard errors, critical ratio and significances.

**Confirmatory factor analysis. Regression Weights, standard errors, critical ratio and significances.**						
**Regression Weights: (Group number 1 - Default model).**		**Estimate**	**S.E.**	**C.R.**	**P.**	**Label**
Item 1.	Usability.	1.000				
Item 2.	Usability.	.895	.078	11.405	***	par_1
Item 3.	Usability.	.927	.072	12.946	***	par_2
Item 4.	Usability.	1.000				
Item 5.	Utility.	.758	.051	14.838	***	par_3
Item 6.	Utility.	.603	.044	13.642	***	par_4
Item 7.	Trust and confidence.	1.000				
Item 8.	Trust and confidence.	1.961	.212	9.272	***	par_5
Item 9.	Well-child section.	1.000				
Item 10.	Well-child section.	0.836	.038	21.791	***	par_6
Item 11.	Well-child section.	0.880	.050	17.670	***	par_7
Item 12.	Well-child section.	0.813	.048	16.876	***	par_8
Item 13.	Accessibility.	1.000				
Item 14.	Accessibility.	0.622	.069	8.980	***	par_9

[Supplementary file A11].

Confirmatory factor analysis. Chi-Square Test.

**Confirmatory factor analysis. Chi-Square Test.**	
**Notes for model. Computation of degrees of freedom (Default model).**	
Number of distinct sample moments.	105
Number of distinct parameters to be estimated.	38
Degrees of freedom (105 - 38).	67
**Result (Default model). Minimum was achieved.**	
Chi-square.	290.438
Degrees of freedom.	67
Probability level.	0.000
Estimates (Group number 1 - Default model).<br>Scalar Estimates (Group number 1 - Default model).<br>Maximum Likelihood Estimates.	

[Supplementary file A12].

Confirmatory factor analysis. Model adjustment measures.

Confirmatory factor analysis. Model adjustment measures.			
**Adjustment measure.**	**Default mode.**	**Saturated mode.**	**Independence model.**
**NFI**	.907	1.000	
**RFI**	.874		0.000
**IFI**	.927	1,000	0.000
**TLI**	.900		0.000
**CFI**	.926	1.000	0.000
**GFI**	.919	1.000	
**AGFI**	.872		
**RMSEA**	.080		.254
**LO 90**	.071		.247
**HI 90**	.090		.262
